# Comparative *in‐vitro* assessment of RV and KI loaded nanostructured lipid carriers (NLCs) individually and in combination on Neuro‐2a cell lines

**DOI:** 10.1002/alz.095298

**Published:** 2025-01-09

**Authors:** Saif Ahmad Khan, Zufika Qamar, Pirthi Pal Singh, Suhel Pervez, Sanjula Baboota, Javed Ali

**Affiliations:** ^1^ Jamia Hamdard, New Delhi, Delhi India; ^2^ Tirupati Group, Paonta Sahib, Himachal Pradesh India

## Abstract

**Background:**

Butyrylcholinesterase enzyme inhibitor (RV), and kinase inhibitor (KI), as combination therapy is considered more effective treatment approach for Alzheimer’s disease (AD). Hence, this combination has been chosen as a treatment strategy. So, this study emphasises the effectiveness of RV and KI in individuals and in combination on the viability of cell lines.

**Method:**

The cytotoxicity of each drug namely RV and KI were screened on Neuro‐2a cell using MTT assay. The treatments of cells were done with different concentrations of individual drug ranging from 0.97 to 500 µg/ml. Afterwards, IC_50_ of RV and KI was established, and ratio of both drugs was optimized using combination index (CI), assessing their impact on cell line viability. So, optimised combination ratio (1:1) of RV and KI were loaded in the NLC which was formulated using modified emulsio‐sonication method and optimized using Central Composite Design. Simultaneously, individual drug was also loaded in the NLC using same method. Later the viability of cell line towards different formulations was studied and compared at different concentrations.

**Results:**

On performing cell line studies, it was observed that there was dose‐dependent %cell viability of drug‐loaded formulation, individually and in combination. At concentration of 1.95 µg/ml, RV‐NLC and KI‐NLC showed there was significant increase *(p<0.05)* i.e., 25 to 30% in the viability of the cells when compared to RV suspension and KI suspension. On other hand, (RV‐KI)‐NLC showed the highest %cell viability at 500 µg/ml in comparison to the other NLC formulations.

**Conclusion:**

RV‐NLC, KI‐NLC and RV‐KI NLC exhibited improved delivery of RV and KI into Neuro‐2a cell lines, resulting in higher viability efficacy in comparison to the suspensions of the drugs. However, (RV‐KI)‐loaded NLCs demonstrated even greater viability of the cell lines than the individual drug‐loaded NLCs. Therefore, dual drug‐loaded NLCs can be used as promising drug delivery system for achieving excellent therapeutic efficacy in the treatment of AD.

